# The lightest organic radical cation for charge storage in redox flow batteries

**DOI:** 10.1038/srep32102

**Published:** 2016-08-25

**Authors:** Jinhua Huang, Baofei Pan, Wentao Duan, Xiaoliang Wei, Rajeev S. Assary, Liang Su, Fikile R. Brushett, Lei Cheng, Chen Liao, Magali S. Ferrandon, Wei Wang, Zhengcheng Zhang, Anthony K. Burrell, Larry A. Curtiss, Ilya A. Shkrob, Jeffrey S. Moore, Lu Zhang

**Affiliations:** 1 Joint Center for Energy Storage Research, Argonne National Laboratory, Argonne, IL 60439, USA; 2 Chemical Sciences and Engineering Division, Argonne National Laboratory, Argonne, IL 60439, USA; 3 Energy & Environmental Directorate, Pacific Northwest National Laboratory, Richland, WA 99352, USA; 4 Materials Science Division; Argonne National Laboratory, Argonne, IL 60439, USA; 5 Department of Chemical Engineering, Massachusetts Institute of Technology, Cambridge, MA 02139, USA; 6 Department of Chemistry, University of Illinois at Urbana-Champaign, Urbana, Illinois 61801, USA; 7 Beckman Institute for Advanced Science and Technology, University of Illinois at Urbana-Champaign, Urbana, Illinois 61801, USA

## Abstract

In advanced electrical grids of the future, electrochemically rechargeable fluids of high energy density will capture the power generated from intermittent sources like solar and wind. To meet this outstanding technological demand there is a need to understand the fundamental limits and interplay of electrochemical potential, stability, and solubility in low-weight redox-active molecules. By generating a combinatorial set of 1,4-dimethoxybenzene derivatives with different arrangements of substituents, we discovered a minimalistic structure that combines exceptional long-term stability in its oxidized form and a record-breaking intrinsic capacity of 161 mAh/g. The nonaqueous redox flow battery has been demonstrated that uses this molecule as a catholyte material and operated stably for 100 charge/discharge cycles. The observed stability trends are rationalized by mechanistic considerations of the reaction pathways.

Redox flow batteries (RFBs) store electric energy in redox-active solutes contained in external tanks^1^,^2^,^3^. The electrodes serve only to enable electron transfer to and from redox-active species in the solutions. In RFBs, energy and power are decoupled and therefore independently scalable according to the application demands. The aqueous RFBs developed to date include iron-chromium, bromine-polysulfide, vanadium-bromine, all-vanadium, and all-organic systems^3^,^4^,^5^,^6^,^7^,^8^. Due to the low cell voltage (ca. 1.5 V), the energy density of aqueous RFBs is low, which in turn requires larger tank volumes to hold more electroactive materials and drives up costs^9^. Non-aqueous solutions enable a wider electrochemical window potentially resulting in a higher energy density and lower cost^10^,^11^. Both transition metal-ligand complexes and redox-active organic molecules (ROMs) have been considered for use in non-aqueous RFBs (NRFBs)^12^,^13^,^14^,^15^,^16^,^17^. The ROMs flowing through the positive and negative compartments of NRFBs are typically different from each other, and these molecules are referred to as the catholyte and anolyte species, respectively^3^,^13^.

The optimum catholyte species must combine high solubility, electrochemical reversibility, high redox potential, and outstanding stability in all states of charge. A desirable property of such materials is high intrinsic capacity, defined as the theoretical amount of charge (in mAh) stored per gram of substance. *The higher the molecular weight, the lower the intrinsic capacity; hence the quest for the low-weight ROMs that achieve all of the above-mentioned criteria.*

Presently, the catholytes developed for NRFBs fall into three broad categories: organometallic compounds, such as ferrocene derivatives^18^, organic radicals, such as (2,2,6,6,-tetramethylpiperidin-1-yl)oxyl (TEMPO)^16^, and closed-shell organic molecules including 1,4-dimethoxybenzene derivatives. While the first two categories yield chemically stable oxidized species, the stability is a trade-off: the radical cations of 1,4-dimethoxybenzenes have much greater specific energy density, as the corresponding redox potentials are considerably higher (by as much as 0.5–1.0 V). However, such energy-rich radical cations also tend to have short lifetimes in room-temperature solutions due to their very reactivity. A key challenge is to find ROMs that are as light as possible (to yield high intrinsic capacity), with radical cations unreactive as possible (for long-term charge storage), and at the same time have high solubility in all states of charge, with a high redox potential. In this study, we report on a discovery of such an organic radical cation.

Common reactions of radical cations are deprotonation, disproportionation, and radical addition involving the parent compound or another radical cation^19^,^20^,^21^. Only a few known radical cations (such as substituted dialkoxybenzene, see below) are stable in liquid organic solvents on the time scales required for electrical energy storage and these tend to require “steric protection” to suppress bimolecular decomposition pathways, such as radical addition in the benzene ring. While such steric protection is an effective strategy for improving stability (e.g., by using bulky *tert*-butyl groups), the resulting compounds often have prohibitively high molecular weight and low solubility in the polar solvents that are used as electrolytes. The inclusion of compensating polar groups is required to increase solubility, but this modification implies lower intrinsic capacity.

Derivatives of 1,4-dimethoxybenzene (compound **1** in Fig. 1) are recognized as a promising class of catholyte materials, as they yield stable radical cations^22^,^23^,^24^. A representative example is 2,5-di-*tert*-butyl-1,4-bis(2-methoxyethoxy)benzene, DBBB (compound **2** in Fig. 1)^23^ that has recently been successfully demonstrated in NRFBs^12^. However, the low solubility and intrinsic capacity (79 mAh/g) of DBBB limit its practical utility. Specifically, the techno-economic model for NRFBs suggests a target capacity of 178 mAh/g for cost-effective widespread adoption^25^. While this intrinsic capacity is desirable, it can be challenging to obtain in practice.

To address this challenge, we applied a molecular “pruning” approach to the DBBB structure. In this approach we systematically shed non-essential groups^26^, which led to an initial set of low-weight 1,4-dimethoxybenzene derivatives shown in Fig. 1, in an effort to find the smallest redox reversible molecule with the lightest steric screen. The synthetic procedures are given in Supplementary Section 1 and spectroscopic data are given Supplementary Figs 1 to 16. In the section that follows, we outline an experimental strategy to develop energy-dense catholytes based on 1,4-dimethoxybenzene derivatives. The strategy includes: (i) electrochemical screening, (ii) chemical characterization of the radical cations, and (iii) redox flow battery testing.

## Results

### Electrochemical Screening

**Figure 1:**
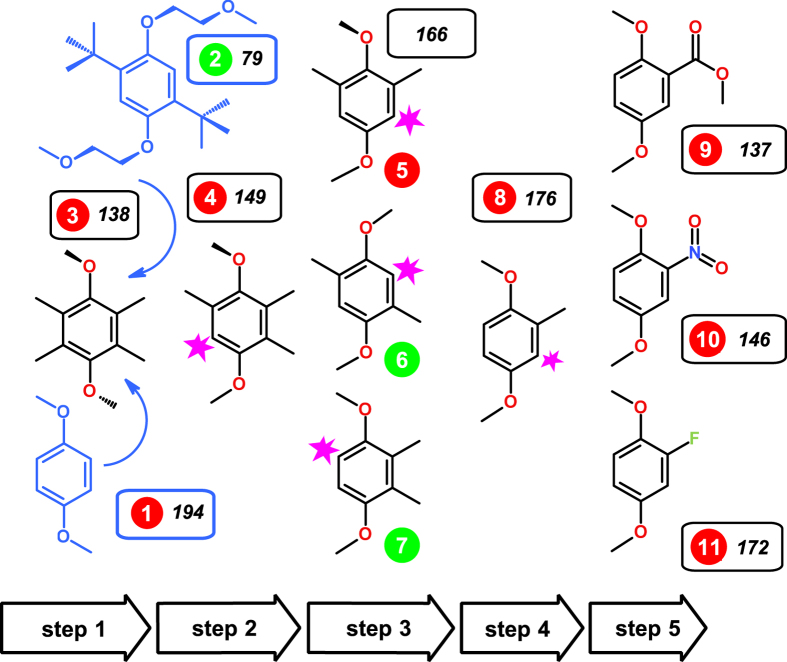
“Molecular pruning” workflow. The stars designate the locations where groups were removed by pruning. The intrinsic capacity of these compounds (in mAh/g) is indicated in the scheme. The green/red circles indicate compounds that were reversibly/irreversibly oxidized in the cyclic voltammetry trials.

**Figure 2:**
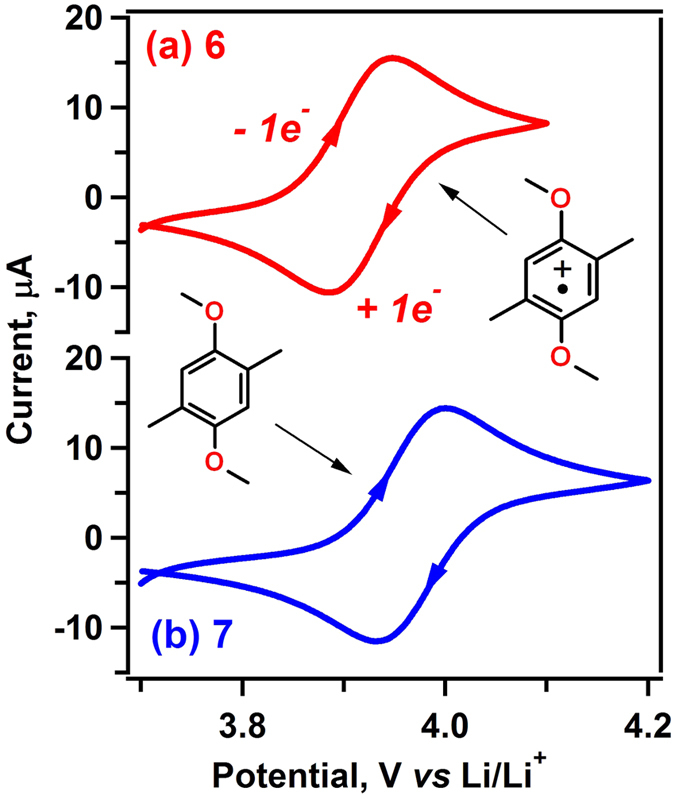
Cyclic voltammetry of 10 mM ROMs in a carbonate electrolyte. (**a**) **6** and (**b**) **7** obtained at the scan rate of 10 mV/s.

In the first stage of screening the electrochemical reversibility was assessed (Table 1, Fig. 2 and Supplementary Figs 17 to 19). This is the step where most compounds shown in Fig. 1 get excluded from further scrutiny. The ROMs **1** to **11** were examined using cyclic voltammetry in Pt/Li/Li three-electrode cells containing 10 mM ROM in 1 M LiTFSI (lithium bis(trifluoromethane)sulfonimide) in a solvent mixture of ethylene carbonate (EC), propylene carbonate (PC), and ethyl methyl carbonate (EMC) in a weight ratio of 4:1:5 (this electrolyte composition has been optimized for the RFB testing)^18^. Using this method, we eliminated the molecules whose radical cations were so reactive that they decayed irreversibly during the electrochemical cycling at 10–100 mV/s (Table 1), implying the lifetimes for their radical cations are certainly less than 1 min. Table 1 gives the redox potentials vs. Li/Li^+^ and (for reversible systems) the ratios *i*_pa_/*i*_pc_ of the currents corresponding to the anodic and cathodic peaks, respectively. Based on these data we ranked the systems as acceptable or not acceptable for further examination.

Consistent with previous studies, the lightest compound **1** is not electrochemically reversible; i.e., some degree of steric protection is required to achieve redox reversibility^19^,^20^,^21^,^27^. Starting from the well-behaved molecule DBBB (compound **2** in Fig. 1)^23^, we used a systematic pruning study beginning with **3** (step 1 in Fig. 1), a compound in which the aromatic ring is fully substituted with four methyl groups. This strategy not only provides an effective way to identify the highest intrinsic capacity of this 1,4-dimethoxybenzene system, but also provides an opportunity for insightful study of the structure-activity relationship of the differently substituted patterns. As seen from Table 1, the electrochemical reactions of **3** were not fully reversible, which makes it unsuitable for further scrutiny. Pruning this congested molecule yields compound **4** (step 2 in Fig. 1) whose oxidation was also not fully reversible. Pruning once more we obtained isobaric compounds **5**, **6**, and **7** (step 3 in Fig. 1). For compound **5**, two oxidation peaks were observed, but the processes are imperfectly reversible; in contrast, compounds **6** and **7** (both of which have the intrinsic capacity of 161 mAh/g) exhibited well-defined, reversible redox waves at 3.92 and 3.98 V as seen in Fig. 2. Given that **1** is not redox reversible, we checked whether further pruning of **6** and **7** leads to a still lighter molecule whose oxidation is reversible. Therefore, we examined compound **8** (step 4 in Fig. 1) and found that it displayed irreversible oxidation.

This systematic pruning approach revealed important and surprising results. First, promising low-weight derivatives were found to exist based solely on methyl group screening. Specifically, we found dimethyl derivatives **6** and **7** were well behaved in cyclic voltammetry. Second, the promising derivatives differ from all the others in subtle and not particularly intuitive ways, reflecting a balance in the degree and placement of methyl screening groups. Although the monomethyl derivative **8** was not promising, it is conceivable that another low molecular weight group is capable of protecting the radical cation. To explore this possibility, compounds **9**, **10**, and **11** with the electron withdrawing groups at the carbon-2 position were synthesized and screened (step 5 in Fig. 1). We intentionally selected electron-withdrawing groups in order to maintain a high oxidation potential and hence a high energy density. The electrochemical oxidation of **9** and **10** was not perfectly reversible especially at a scan rate of 10 mV/s and their relatively high molecular weight (compared to **6** and **7**) was not conducive to our goal of finding the lightest molecule of highest capacity. Thus, we synthesized **11**, as the fluorine atom is the lightest electron-withdrawing group. If one-electron oxidation of **11** were reversible, it would yield the highest intrinsic capacity of 172 mAh/g, but, unfortunately, **11** displayed redox behavior similar to **8**.

In Supplementary Section 2 and Figs 20 to 23 we demonstrate that the rapid decay of radical cations in our cyclic voltammetry tests (with the exception of **6** and **7**) that caused irreversibility was due to their efficient deprotonation in the carbonate solvent. This deprotonation was sufficiently slow to justify further scrutiny only for **6** and **7**.

### Chemical Properties of the Radical Cations

As the radical cations used in the NRFBs need to hold charge over many hours of operation, we assessed the long-term chemical stability of radical cations of **6** and **7** (denoted in the following as **6**^+∙^ and **7**^+∙^). To simplify spectroscopic characterization and product analyses, a simpler electrolyte composition, viz. PC containing 0.5 M LiTFSI, was used. We chose a relatively low concentration of ROMs (1 mM) to determine what limits the *inherent* chemical stability of their radical cations (in high concentration regimes second order reactions can prevail).

**Figure 3:**
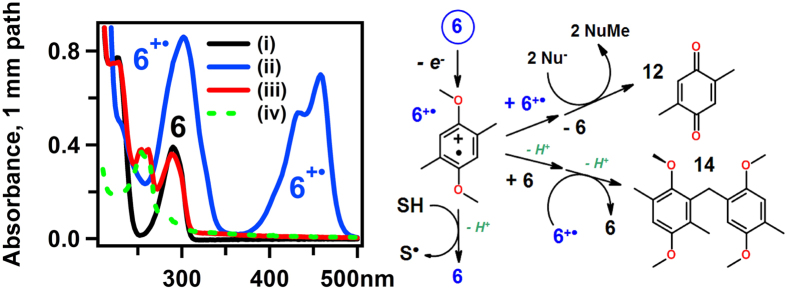
The absorption spectra of 6 following electrochemical oxidation and a sketch of the unfolding radical cation chemistry. *To the left:* Trace i was obtained before the electrolysis, trace ii was obtained immediately after the electrolysis, trace iii was obtained after the decay of **6**^+∙^, and trace iv is the difference traces obtained as explained in the text. *To the right:* reactions of **6**^+∙^ leading to ROM recovery (via deprotonation with the subsequent H abstraction from the solvent, SH), the formation of quinone **12** (via radical disproportionation and *O*-demethylation to a nucleophile Nu^−^ in solution) and ring-to-methyl dimer **14** (via radical addition).

A combination of several spectroscopic techniques was used to identify the reaction products following electrochemical oxidation of ROMs to 90% state-of-charge. In UV spectroscopy, the parent compounds have strong absorption bands at 290 nm (trace i in Fig. 3 and Supplementary Fig. 24). Following electrochemical oxidation of 1 mM solutions of 6 or 7 in the selected electrolyte, the absorbance bands of the radical cations are observed (trace ii in Fig. 3). These absorption spectra are similar to the ones reported for **1**^+∙^^20^,^21^. Over several days, the absorbance of the radical cation gradually fades and the absorbance of the parent compounds increases, suggesting the occurrence of one-electron reduction. The residual absorbance (trace iii in Fig. 3) is comprised of the spectral bands of the parent compound that strongly overlap with the bands of a new species with the absorption maxima at 250–260 nm. The recovery yield of the ROMs was determined by NMR (Supplementary Fig. 25), and the appropriately weighed trace i was subtracted from trace iii in Fig. 3 (for **6**^+∙^) to obtain trace iv, which corresponds to the absorbance of reaction products. This spectrum is strikingly similar to that for 2,5-dimethylbenzoquinone **12** (see also Supplementary Fig. 26), which is the product of two-electron oxidation of **6** (see Fig. 3). The same species is also the product of radical disproportionation (Fig. 3) that occurs through the loss of two methyl groups from a reaction intermediate to nucleophiles present in solution (Nu^−^ in Fig. 3)^28^. The formation of this quinone is demonstrated in Supplementary Fig. 27.

To quantify the reaction yield, high performance liquid chromatography (HPLC) was used. In the chromatograms obtained immediately after the electrolysis (Supplementary Figs 28 and 29), there are two main peaks, one of which corresponds to the parent compound and the other to the corresponding quinone. It was found that some two-electron oxidation occurred already during the electrolysis, yielding ~5% of the quinones for both **6** and **7**. Over time, as the radical cation decays, the yield of the quinone increases to 13–17%. This result suggests that radical cations in carbonate solvents undergo disproportionation, yielding the parent compound and the quinone (Fig. 3).

In addition, some other reaction products were generated with a low yield. In particular, *m/z* 330 dimers for both **6** and **7** were observed (see Supporting Section 3 and Figs 30 to 32 and Tables 1 and 2 therein). The yield of these dimers was ca. 2–3%, and the yields of other volatile products were even lower (<0.5%). The dimers originate through addition of the radical cations to their parent compounds as shown in Fig. 3^19^,^20^. In more concentrated solutions and/or other organic solvents, the yield of the dimer for **7** can significantly increase, while that for **6** always remains low. For example, in electrolysis of 5 mM solutions of **6** and **7** in acetonitrile containing 0.5 M LiPF_6_, the yields of the dimers were 1% and 22%, respectively. In Supporting Section 3 we demonstrate that ring-to-ring bridging (**13** as seen in Supplementary Section 3) occurs for **7**^**+**∙^ while only methyl-to-ring bridging (**14** in Fig. 3) occurs for **6**^**+**∙^ Apparently, 2,3-dimethyl substitution still leaves **7**^+∙^ vulnerable to ring-to-ring addition of the radical cation to the parent compound, whereas 2,5-dimethyl substitution entirely excludes such a reaction.

**Figure 4:**
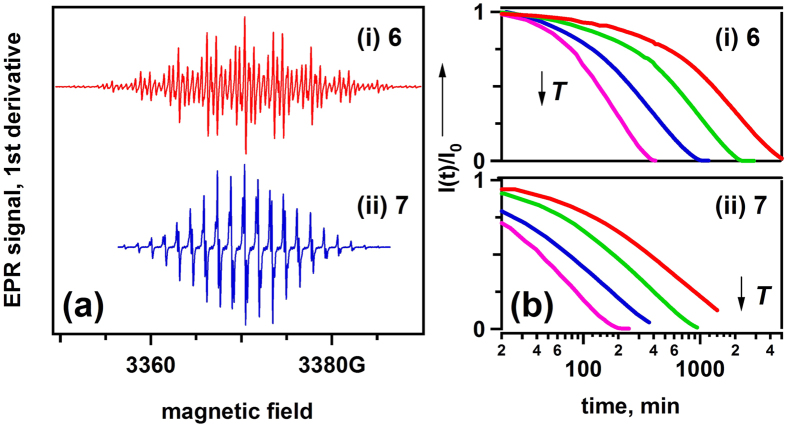
EPR observations of ROMs. (**a**) First-derivative continuous wave EPR spectra (1 G = 10^−4^ T, 0.5 G modulation at 100 kHz) and (**b**) decay kinetics for the normalized doubly integrated EPR signals *I(t)* of (i) **6**^+∙^ and (ii) **7**^+∙^ observed in electrochemically oxidized 1 mM solutions. These kinetics were obtained at T = 21.7, 32.5, 41.3 and 57.0 °C. *I*_0_ in panel b refers to *I*(t = 0). Mind the logarithmic time scale.

To better characterize the structure and stability of these radical cations, Electron Paramagnetic Resonance (EPR) spectroscopy was used. The first-derivative EPR spectra for **6**^+∙^ and **7**^+∙^ cations were obtained (Fig. 4a and Supplementary Fig. 33), and their magnetic parameters were determined and compared with the estimates obtained from quantum chemistry calculations (Supplementary Table 3). This comparison indicates that only one type of paramagnetic species is present in the solution at all times, which are **6**^+∙^ and **7**^+∙^. Figure 4b shows the decay kinetics of the normalized integrated EPR signals. As seen from these plots, **6**^+∙^ is significantly more stable than **7**^+∙^ in all of the temperature ranges. The initial decay of **6**^+∙^ is (pseudo)first order (Supplementary Fig. 34); as the reaction progresses further, this decay accelerates due to reactions involving the secondary reaction products. Using only the initial decay segments, the activation energy for the decay of **6**^+∙^ is ~13 kcal/mol (Supplementary Fig. 35). It is this high activation energy that is responsible for the strong temperature dependence observed in Fig. 4b. For **7**^+∙^, the decay kinetics is biexponential. While the slow component has an activation energy similar to **6**^+∙^, the fast component has different activation energy (Supplementary Fig. 36). Apparently, **7**^+∙^ accesses a reaction channel (e.g., ring-to-ring addition) that is inaccessible to **6**^+∙^, which makes it less stable.

Based on our results and mechanistic considerations in Supplementary Section 2, in Fig. 3 we summarize the three major reaction channels for **6**^+∙^ decay that include (i) deprotonation with the subsequent H abstraction from the solvent to recover **6**, (ii) radical disproportionation, and (iii) radical addition. Only the first reaction channel leads to the ROM recovery, whereas in other two channels the material is consumed irreversibly. Considering the recovery yields (73% for **6** and 66% for **7**, see Supplementary Fig. 25) it is fair to say that for both **6** and **7**, most of the radical cations convert back to their parent compounds. For **6** (but not for **7**) the radical addition remains inefficient even in more concentrated solutions. As bimolecular reactions of radical cations become increasingly important in the high-concentration regimes that occur in NRFBs, this vulnerability of **7** translates into inferior electrochemical performance.

### Solubility Testing

To further assess the suitability of **6** and **7** as catholyte molecules for NRFB testing, their solubility in 0.5 M LiTFSI in PC at 35 °C was determined, yielding 0.6 M and 2.0 M for **6** and **7**, respectively (compared to just 0.3 M for 2). The improved solubility further justifies the use of these compounds in NRFBs. For compound **6**, the conductivity and viscosity, two important factors affecting the performance of RFBs, were evaluated as a function of the solute concentration and found to remain almost constant at different concentrations of the solute (Supplementary Table 4).

### Flow Cell Testing

An ideal flow battery system features high cell efficiencies and good cycling stability over extended charge/discharge cycling. Coulombic efficiency (CE), voltaic efficiency (VE) and energy efficiency (EE) are defined as the ratios of the capacity, voltage and energy during discharge over those during charge, respectively. The CE characterizes the extent of self-discharge and side reactions while the VE reflects the cell over potential. Flow batteries are generally required to have long calendar and cycle lives. Thus, good cycling stability is critically important for a flow battery system to provide reliable service output and reduce the necessity of electrolyte maintenance.

**Figure 5:**
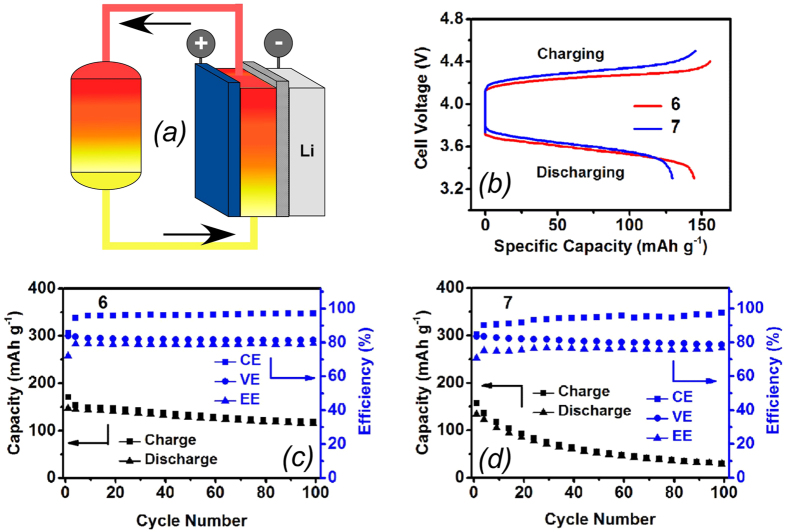
Electrochemical cycling performance tests. (**a**) Schematic drawing of redox flow cells with a graphite cathode and a Li-graphite hybrid anode operating at 7.5 mA/cm^2^. (**b**) The typical charge/discharge voltage curves for these cells containing 0.1 M **6** (red) and **7** (blue). Cycling efficiencies *(in blue, to the right)* and specific capacities *(in black, to the left)* over 100 cycles for (**c**) Li/**6** and (**d**) Li/**7** cells on charge and discharge. CE, VE, and EE are the Coulomb, voltaic, and energy efficiencies.

The electrochemical cycling performance of **6** and **7** was assessed using a lithium-graphite hybrid anode (Fig. 5a)^16^,^18^. The representative charge/discharge voltage curves at a current density of 7.5 mA/cm^2^ are shown in Fig. 5b (see Supplementary Fig. 37 for volumetric energy densities). The charge plateaus indicate formation of radical cations, while the discharge plateaus correspond to their reduction. The flow cells delivered a specific capacity of 145 mAh/g for **6** and of 130 mAh/g for **7**. These values correspond to 90% and 81%, respectively, of the theoretical capacity, indicating high utilization ratios of both **6** and **7** in flow cells. As shown in Fig. 5c, the Li/**6** flow cell produced constant cycling efficiencies throughout 100 cycles, with CE of 96%, VE of 82% and EE of 79%. The high CE is ascribed to the solid electrolyte interphase layers formed on the Li and graphite surfaces that allow only Li^+^ cations to transport across these layers while blocking crossover of redox species. Also, with an average fading rate of 0.2% per cycle, the cycling capacity of the flow cell is relatively stable, reflecting the good chemical stability of **6**^**+**∙^ in the flow cell. Note that the stability of **6**^**+**∙^ showed here is comparable with the anthraquinone system, in which no reactive radical ion was involved^6^. In addition, the Li/**6** flow cell exhibits a great rate capability yielding similar EEs (79%) at both 5.0 and 7.5 mA/cm^2^ (see Supplementary Fig. 38). In contrast, the Li/7 flow cell yielded lower CE (90%) and much faster capacity fading (only 22% of the initial capacity remaining after 100 cycles) under the same flow cell conditions (Fig. 5d), which indicates considerable irreversible side reactions existing for **7**^**+**∙^ during the cycling. This result agrees well with the EPR study demonstrating that **7**^**+**∙^ is less stable. Thus, both the EPR study and the flow cell test results indicate that **6** is a more promising redox material candidate than **7**. Importantly, the capacity loss observed in Fig. 5c,d only partially results from chemical instability of the catholyte species: continuous crossover of redox active molecules across the microporous membranes and gradual increase in the solid electrolyte interphase thickness as Li metal reacts with the solvent and redox active materials are two additional factors causing this capacity loss.

We note that the concentration of ROMs used in flow cells has a significant effect on the cell performance. In addition to the 0.1 M Li/ **6** cell examined above (with the demonstrated volumetric energy densities for charge and discharge of 12 and 9 Wh/L, respectively), we also tested 0.2 M Li/**6** cell (Supplementary Fig. 39) and obtained almost double of these densities (23 and 16 Wh/L for charge and discharge, respectively). The volumetric energy density at 0.2 M **6** is comparable to the one achieved in the Fe/Cr and Fe/V aqueous RFB prototypes at a much greater concentration of the redox materials (>1.25 M)^29^,^30^, indicating the advantages of NRFBs.

## Discussion

In this study, we screened the lightest derivatives of 1,4-dimethoxybenzene, selecting the compounds showing fully reversible electrochemical oxidation, and identified two of them as the most promising candidates. With an appropriate degree of substitution and proper placement, we demonstrated that methyl substitution was sufficient to provide the steric protection against the radical addition reactions while impeding rapid deprotonation of the radical cations caused by congestion in the benzene ring, which leads to localization of positive charge in the out-of-plane methoxy group (see Supplementary Section 2). Compounds **6** and **7** represent the optimum between these two trends, but **7** is still vulnerable to (slow) ring addition of the radical cation to the parent molecule. While this reaction is quite inefficient in dilute solutions, in high-concentration regimes that are more relevant to RFB performance it makes **7** inferior to **6**. In dilute solutions, the radical cations of **6** and **7** are slowly converted back to their parent compounds. The test performance of **6** is comparable to that of the much heavier molecule **2**, and it offers advantages due to the improved solubility and specific capacity. The electrochemical cycling performance of **6** was evaluated using lithium/organic flow cells that yielded high cell efficiencies and good capacity retention.

Our examination sets the upper limit on the intrinsic capacity that can be realistically achieved for 1,4-dimethoxybenzene derivatives given the constraints that are imposed by their structures, chemical reversibility, and the stability of their radical cations. Intriguingly, the intrinsic capacity of 178 mAh/g suggested by Darling *et al*.^25^ as the threshold for techno-economic practicability of a catholyte material is close to 161 mAh/g that was obtained for **6**. Further increase of the intrinsic capacity is likely to require the involvement of reversible two-electron reactions.

The best catholyte ROM is only 2 a.m.u. heavier than tetramethyl-*p*-phenylenediamine (Wurster’s blue) that has long served as the gold standard of stable radical cations in organic chemistry. However, Wurster’s blue has an undesirably lower redox potential (3.00 V) than **6** (3.92 V), which makes **6** superior in terms of the energy density. There is also 2,5-difluoro-1,4-dimethoxybenzene (that is 8 a.m.u. heavier than **6**) for which the reversible oxidation at 4.30 V vs. Li/Li^+^ has been reported, but the operation at this high voltage results in the gradual damage of positive electrode, as this ROM becomes decomposed, releasing the fluoride^31^. Together, these three molecules triangulate the minimum molar mass of a closed shell neutral organic molecule that can be considered for use as a catholyte material, and we argue that **6** offers practical advantages, given the operational constraints of solubility, stability and availability. It presently appears that this compound is the simplest ROM with the required combination of physicochemical and electrochemical properties.

## Methods

### Materials

Lithium ribbon (99.9%, 0.75 mm thick), LiTFSI (99.95%), PC (anhydrous, 99.7%), 1,4-dimethoxybenzene (**1**) and 2-methyl-1,4-dimethoxybenzene (**8**) were obtained from Sigma-Aldrich. LiTFSI was dried in the vacuum oven at 70 °C for 12 h before use. All starting materials and reagents were purchased from commercial sources (Sigma-Aldrich, Acros Organics and Alfa Aesar) and used as received. Materials used for flow cell test were purchased from BASF and used as received. Compounds other than **1** and **8** were synthesized by following the procedures described in Supplementary Section 1.

### Cyclic voltammetry

Cyclic voltammetry for 10 mM ROM was performed with 90% iR compensation in a custom-made three electrode cell with a 2 mm diameter platinum working electrode, a Li metal (pseudo)reference electrode, and a Li metal counter electrode. The data were collected on a CHI660D Potentiostat (CH Instruments) in an Ar-filled glove box at 30 °C.

### Radical cation generation

For chemical oxidation, 25 mmol [bis(trifluoroacetoxy)iodo] benzene was added to 5 mmol ROM in 5 mL butyronitrile containing 25 mmol trifluoroacetic acid. Alternatively, the compounds were introduced in a solid form into 70% aqueous sulfuric acid, where they are simultaneously dissolved and oxidized. For electrochemical oxidation, we used constant potential bulk electrolysis with 1 mM ROM in 0.5 M LiTFSI in PC; in some experiments we used 5 mM ROM and replaced PC by acetonitrile. The voltage was set at 4.15 V and the state of charge was 90%.

### EPR spectroscopy

100 μL aliquots of oxidized liquid samples were placed in graduated capillaries and flame sealed in glass tubes. The first-derivative EPR spectra were collected using a Bruker ESP300E X-band spectrometer. Temperature controlled nitrogen gas flow was used to stabilize the sample temperature. The analysis of the EPR spectra was carried out using WinSim program suite (v. 0.98). For kinetic analyses, the EPR spectra were filtered using the initial spectrum as a mask and then doubly integrated.

### Chemical characterization

The absorption spectra were obtained using an Olis Cary 14C spectrometer in dry N_2_ atmosphere in a 1 mm optical path quartz cell. For high performance liquid chromatography isocratic acetonitrile elution at 0.25 mL/min was used. 5 μL of the sample was loaded on a Supelco LC-PAH column (length 25 mm, bore 4.6 mm, particle size 5 μL), and the absorbance was detected using a photodiode array detector using a ThermoScientific Accela suit. Electrospray ionization mass spectra were obtained using the direct injection of dilute sample using the same setup. For gas chromatography-mass spectrometry measurements, 1 μL liquid sample was loaded in a splitless mode on an HP-5MS (bore 0.25 μm, length 30 m) column using an Agilent Technologies Model 7890B chromatograph equipped with a Model 5977 mass detector. To remove lithium salt from the electrolyzed solutions, the samples for GCMS were diluted 1:100 v/v with dichloromethane, centrifuged at 3000 ppm and the supernatant was separated and reduced in vacuum. ^1^H NMR spectra were obtained using an Avance DMX 500 MHz spectrometer (Bruker). To concentrate the reaction products in PC for further identification, the reaction mixture was diluted with an excess of water (1:100 v/v), the products were absorbed on Hypersep C18 packed column, the co-adsorbed PC was washed with excess of water, the adsorbed products were stripped with methanol, and the organic solvent was removed in vacuum. This treatment allowed us to identify the mass peak of the quinones using GCMS and also observe the resonance lines of these products using ^1^H NMR spectroscopy. To analyze the acetonitrile solutions, the solvent was removed in vacuum, *n*-hexane was added to extract reaction products, and the solvent was once again removed in vacuum and replaced with acetonitrile. The resulting mixture was chromatographically separated, the acetonitrile in eluted fractions was removed in vacuum, and GCMS chromatograms and ^1^H NMR spectra for each major reaction product were obtained. The chemical structures of compounds **13** and **14** were established in this manner (see Supplementary Section 3 for more detail).

### Conductivity, viscosity, and solubility measurements

Impedance spectroscopy was used to measure the ionic conductivity of the electrolytes containing ROMs. The house-made dip-in conductivity cell consisting of two platinum disks was used. Viscosities were measured using a VISCOlab4000 Laboratory Viscometer (Cambridge Viscosity). The sample chamber was thermostated at 30 °C during the measurements (see Supplementary Table 2). For solubility measurements at 35 °C, samples containing different concentration of ROMs were transferred into a 96-well quartz plate after being stirred at this temperature in sealed vials for 1 hour. They were then investigated using a dynamic light scattering analyzer equipped with an optical camera and a temperature controller (DynaPro Plate Reader II, Wyatt Technologies). The assessment was performed using the images of each well.

### Flow battery testing

The electrode compartments of the flow cells involved a hybrid anode, a graphite felt (GFD3 EA grade, 3 mm thick, 300 g/m^2^; SGL Group) at the cathode side, and a polyethylene-based microporous separator (800 μm thick, 60% porosity, 0.1–1 μm pore; Daramic) sandwiched in between (Fig. 5a). The hybrid anode consisted of directly stacked Li foil and graphite felt strip. Prior to use, the separator and the graphite felt were vacuum dried at 70 °C for 24 h to remove moisture. The active area of the flow cell was 2 cm wide and 20 cm long. Unless stated otherwise, the electrolyte composition was 0.1 M **6** or **7** in 1 M LiTFSI in EC/PC/EMC (4:1:5 by weight) containing 5 wt% fluoroethylene carbonate to protect the anode. Some cycling tests were also performed using 0.2 M **6**. Each cycle lasted ca. 30 min. The hybrid anode was saturated with a static electrolyte (11 mL) that was not included in calculating the specific capacity or energy density. This static solution had the same composition as the catholyte, which greatly reduced the concentration gradient and crossover of ROM species across the polymer separator. A flowing electrolyte (14 mL) was circulated through the graphite felt cathode by using a Masterflex L/S peristaltic pump (Cole-Parmer) at a flow rate of 50 mL/min. The flow cell charge/discharge cycling was performed on an Arbin BT-2000 battery tester housed in an Ar-filled glove box at 30 °C. The calculation of specific capacity was based on the cycled capacity divided by the mass of redox materials in the catholyte. The volumetric energy density was calculated through the cycled energy divided by the volume of the catholyte.

## Additional Information

**How to cite this article**: Huang, J. *et al*. The lightest organic radical cation for charge storage in redox flow batteries. *Sci. Rep.* **6**, 32102; doi: 10.1038/srep32102 (2016).

## Supplementary Material

Supplementary Information

## Figures and Tables

**Table 1:** Comparison of the performance for compounds 1 to 11 by cyclic voltammetry tests^a^.

No.	MW^b^	*E*_1_^0^(V)^c^	*E*_2_^0^(V)^c^	*i*_pa_/*i*_pc_^d^	Acceptable?
1	138	—	—	—	no
2	338	3.94	—	1.07	yes
3	194	—	—	—	no
4	180	4.07	—	2.11	no
5	166	4.10	4.26	—	no
6	166	3.92	—	1.04	yes
7	166	3.98	—	1.08	yes
8	152	3.96	4.14	—	no
9	196	4.30	—	1.46	no
10	183	4.48	—	1.33	no
11	156	4.17	4.35	—	no

^a^1 M LiTFSI in EC/PC/EMC (4:1:5 by weight);

^b^Molecular weight in a.m.u.;

^c^The first and second redox potentials vs. Li/Li^+^;

^d^The ratio of the anodic and cathodic peak currents at the voltage scan rate of 10 mV/s.
