# Supplementary material for: The lightest organic radical cation for charge storage in redox flow batteries
Source: Sci Rep. 2016 Aug 25;6:32102. doi: 10.1038/srep32102 (PMC4997354; doi:10.1038/srep32102)
Supplement: Supplementary Information [file srep32102-s1.docx]

Supplementary Information

The lightest organic radical cation for charge storage in redox flow batteries.

Jinhua Huang, Baofei Pan, Wentao Duan, Xiaoliang Wei, Rajeev S. Assary, Liang Su, Fikile R. Brushett, Lei Cheng, Chen Liao, Magali S. Ferrandon, Wei Wang, Zhengcheng Zhang, Anthony K. Burrell, Larry A. Curtiss, Ilya A. Shkrob, Jeffrey S. Moore, and Lu Zhang

**Alphabetical list of abbreviations**

B3LYP Becke, 3-parameter, Lee-Yang-Parr functional

CE Coulombic efficiency

CV cycling voltammetry

EC ethylene carbonate

EE energy efficiency

EMC ethyl methyl carbonate

EPR electron paramagnetic resonance

DBBB 2,5-di-*tert*-butyl-1,4-bis(2-methoxyethoxy)benzene

HPLC high performance liquid chromatography

GCMS gas chromatography – mass spectrometry

LiTFSI lithium bistriflimide, LiN(SO_2_CF_3_)_2_

NRFB non-aqueous RFB

NMR nuclear magnetic resonance

OD optical density

PC propylene carbonate

RFB redox flow battery

ROM redox active organic molecule

TEMPO (2,2,6,6,-tetramethylpiperidin-1-yl)oxyl radical

TFSI N(SO_2_CF_3_)_2_ anion

TMS tetramethylsilane

VE voltaic efficiency

**Supplementary Section 1.** Synthetic procedures.

Compounds **3-7** and **9-11** were synthesized by following the two typical synthetic procedures:

*Procedure I* (compound **6**): To a solution of 2,5-dimethyl-1,4-benzoquinone (13.62 g, 100 mmol) in ethanol (100 mL) and water (100 mL) was added NaBH_4_ (7.57 g, 200 mmol) in small portions under nitrogen. The reaction was stirred at room temperature for 30 min, then a solution of KOH (40 mL, 10 M, 400 mmol) was added and the resulting mixture was stirred for another 30 min, then it was heated to reflux, and dimethyl sulfate (37.85 mL, 400 mmol) was added dropwise. The resulting mixture was stirred at reflux for 12 h before it was cooled to room temperature and extracted with ethyl acetate. The combined organic layers were washed with water and dried over Na_2_SO_4_. After concentration *in vacuo*, the residue was purified by flash column chromatography (silica gel, eluted with ethyl acetate/hexanes=1/10) to yield **6** (14.57 g, 88%) as a white solid. ^1^H NMR (500 MHz, CDCl_3_) δ 6.68 (s, 2H), 3.80 (s, 6H), 2.23 (s, 6H); ^13^C NMR (125 MHz, CDCl_3_) δ 151.4, 124.3, 113.7, 56.1, 16.1.

*Procedure II* (compound **7**)*:* To a solution of 2,3-dimethylhydroquinone (54.5 g, 0.4 mol) in anhydrous *N,N’-*dimethylformamide (400 mL) was added NaH (35.2 g, 60% dispersion in oil, 0.88 mol) in small portions under nitrogen. After the gas evolution had ceased, the resulting mixture was stirred at room temperature for 30 min. The iodomethane (99.6 mL, 1.6 mol) was added dropwise to this reaction mixture, which was stirred at 40 ^o^C for 2 h. Brine was added slowly to quench the reaction and the resulting mixture was extracted with ethyl acetate. The combined organic layers were washed with water and dried over Na_2_SO_4_. After concentration *in vacuo*, the residue was purified by flash column chromatography (silica gel, eluted with ethyl acetate/hexanes=1/20) to yield **7** (63.08 g, 95%) as a white solid. ^1^H NMR (500 MHz, CDCl_3_) δ 6.67 (s, 2H), 3.78 (s, 6H), 2.17 (s, 6H); ^13^C NMR (125 MHz, CDCl_3_) δ 151.9, 126.7, 107.9, 56.1, 12.0.

2,3,5,6-Tetramethyl-1,4-dimethoxybenzene **(3)** was prepared from duroquinone (2.46 g, 15 mmol) following procedure I. Using ethyl acetate/hexanes=1/10 v/v as eluent, compound **3** (2.16 g, 74%) was isolated as a white solid. ^1^H NMR (500 MHz, CDCl_3_) δ 3.64 (s, 6H), 2.18 (s, 12H); ^13^C NMR (125 MHz, CDCl_3_) δ 153.0, 127.9, 60.4, 12.8.

 2,3,6-Trimethyl-1,4-dimethoxybenzene **(4)** was prepared from trimethylhydroquinone (1.52 g, 10 mmol) following procedure II. Using ethyl acetate/hexanes=1/20 v/v as eluent, compound **4** (1.75 g, 97%) was isolated as a colorless liquid. ^1^H NMR (500 MHz, CDCl_3_) δ 6.55 (s, 1H), 3.79 (d, *J* = 0.6 Hz, 3H), 3.67 (d, *J* = 0.8 Hz, 3H), 2.30 (s, 3H), 2.22 (s, 3H), 2.13 (s, 3H); ^13^C NMR (125 MHz, CDCl_3_) δ 153.5, 150.6, 130.6, 127.7, 123.8, 110.3, 60.1, 55.8, 16.3, 12.6, 11.9.

2,5-dimethyl-1,4-dimethoxybenzene **(5)** was prepared from 2,6-dimethylbenzoquinone (0.68 g, 5 mmol) following procedure I. Using ethyl acetate/hexanes=1/15 v/v as eluent, compound **5** (0.73 g, 88%) was isolated as a colorless liquid. ^1^H NMR (500 MHz, CDCl_3_) δ 6.55 (s, 2H), 3.75 (s, 6H), 3.68 (s, 3H), 2.27 (s, 3H); ^13^C NMR (125 MHz, CDCl_3_) δ 155.3, 150.8, 131.7, 113.7, 59.9, 55.4, 16.3.

 Methyl 2,5-dimethoxybenzoate **(9)** was prepared from methyl 2,5-dihydrobenzoate (1.68 g, 10 mmol) following procedure II. Using ethyl acetate/hexanes=1/2 v/v as eluent, compound **9** (1.86 g, 95%) was isolated as a yellow liquid. ^1^H NMR (500 MHz, CDCl_3_) δ 7.32 (d, *J* = 3.2 Hz, 1H), 7.01 (dd, *J* = 9.1, 3.2 Hz, 1H), 6.91 (d, *J* = 9.1 Hz, 1H), 3.88 (s, 3H), 3.85 (s, 3H), 3.78 (s, 3H); ^13^C NMR (125 MHz, CDCl_3_) δ 166.5, 153.5, 153.0, 120.5, 119.6, 116.0, 113.9, 56.8, 55.8, 52.1.

 3,5-Dimethoxynitrobenzene (**10**): To a solution of 4-methoxy-2-nitrophenol (1.69 g, 10 mmol) in anhydrous *N,N’*-dimethylformamide (50 mL) was added NaH (480 mg, 60% dispersion in oil, 12 mmol) in small portions under nitrogen. After the gas evolution had ceased, the resulting mixture was stirred at room temperature for 30 min. The iodomethane (1.25 mL, 20 mmol) was added dropwise to this reaction mixture, which was stirred at 40 ^o^C for 2 h. Brine was added slowly to quench the reaction and the resulting mixture was extracted with ethyl acetate. The combined organic layers were washed with water and dried over Na_2_SO_4_. After concentration *in vacuo*, the residue was purified by flash column chromatography (silica gel, eluted with ethyl acetate/hexanes=1/5) to provide **10** (1.72 g, 94%) as a yellow solid. ^1^H NMR (500 MHz, CDCl_3_) δ 7.40 (d, *J* = 3.1 Hz, 1H), 7.11 (dd, *J* = 9.2, 3.1 Hz, 1H), 7.03 (d, *J* = 9.2 Hz, 1H), 3.92 (s, 3H), 3.82 (s, 3H); ^13^C NMR (125 MHz, CDCl_3_) δ 152.9, 147.4, 139.6, 120.9, 115.2, 110.0, 57.1, 56.1.

2-fluoro-1,4-dimethoxybenzene **(11)** was prepared from fluorohydroquinone (1.28 g, 10 mmol) following procedure II. Using ethyl acetate/hexanes=1/5 v/v as eluent, compound **11** (1.51 g, 97%) was isolated as a colorless liquid. ^1^H NMR (500 MHz, CDCl_3_) δ 6.89 (t, *J* = 9.3 Hz, 1H), 6.69 (dd, *J* = 12.8, 2.9 Hz, 1H), 6.61-6.58 (m, 1H), 3.84 (s, 3H), 3.75 (s, 3H); ^13^C NMR (125 MHz, CDCl_3_) δ 154.0 (d, *J*=9.4 Hz), 152.8 (d, *J*=245.6 Hz), 141.7 (d, *J* = 11.0 Hz), 114.6 (d, *J* = 3.2 Hz), 108.5 (d, *J* = 3.5 Hz), 103.3 (d, *J* = 21.6 Hz), 57.1 (s), 55.7 (s).

**Supplementary Section 2.** Deprotonation as the limiting factor for radical cation stability.

The common chemical reaction responsible for rapid decay of organic radical cations is their deprotonation (Supplementary Fig. 20). The deprotonated species (neutral radicals) subsequently abstract H from the solvent yielding back the parent ROMs, thereby completing the cycle, as shown schematically in Supplementary Fig. 20. Below we argue that (with the exception of **6** and **7**) this reaction shortened the life time of oxidized low-weight ROMs shown in Fig. 1, so that their radical cations decayed on the time scale of cycling voltammetry (CV) tests (< 1 s).

This can be demonstrated by shifting the protonation equilibrium using strong acid. All compounds in Fig. 1 readily dissolve in concentrated sulfuric acid (70 wt% aqueous solution). The solutions become intensely colored as the sulfuric acid oxidizes ROMs, and the formation of the corresponding radical cations can be demonstrated by EPR and optical spectroscopy, as illustrated in Supplementary Fig. 21. The radical cations of **6** and **7** remain stable for over one month in such solutions. Moreover, under such strongly acidic conditions, even radical cations (denoted with ^+●^ in the following) of compounds **3**, **4**, and **5** (that were all unstable in the carbonate solvents in our CV experiments) become stable for hours, suggesting that *rapid deprotonation of these radical cations in carbonate solvents was the cause for their irreversible electrochemical oxidation*. Indeed, when PC was added to these sulfuric acid solutions, the radical cations of **3**, **4**, and **5** rapidly decayed (< 5 s), whereas the radical cations of **6** and **7** persisted for many hours. This can be observed by fading of the absorption bands from the radical cations. For **6**^+●^ (which has intense green coloration) this simple and useful test can be used to assess solvent effects on the radical cation lifetime (see Supplementary Fig. 21). E.g., the color fade is almost immediate in protic solvents like methanol, whereas it is relatively slow in aprotic solvents like acetonitrile and PC, and even slower in weak Lewis acids, such as 2,2,2-trifluoroethanol.

To understand the mechanistic causes for this behavior, density functional theory (DFT) calculations were carried out to study conformations of the parent molecules and radical cations for ROMs shown in Fig. 1. As shown in Supplementary Fig. 22, in the conformationally locked radical cations of **6** and **7**, the methoxy groups are equivalent by symmetry and occupy the plane of the aromatic ring (the deviation is < 20^o^ at 300 K). In **3**^+●^, **4**^+●^ and **5**^+●^, due to steric hindrance from the two adjacent methyl groups, one of these methoxy groups is forced out of plane, and the two methoxy groups become inequivalent, one holding more positive charge than another (Supplementary Figs. 22 and 23). The deprotonation from this electron-deficient methoxy group becomes much more facile.

While insufficient methylation in the benzene ring makes the molecule vulnerable to radical addition (Supplementary Section 3), the *excessive derivatization in the ring makes the radical cation vulnerable to rapid deprotonation by forcing the excess positive charge into one of the methoxy groups*. Compounds **6** and **7** straddle the optimum between these two extremes.

We attribute the somewhat greater stability of **6** with respect to **7** to a greater steric protection in the former molecule, as suggested by our observation of ring-to-ring addition for **7** (see Supplementary Section 3).

**Supplementary Section 3.** Radical addition.

To characterize the products of radical addition for **6** and **7**, we used electrochemical oxidation (100% state-of-charge) of 5 mM solutions in CH_3_CN containing 0.5 M LiPF_6_ (5 mL). Unlike PC, this solvent has only one proton NMR line that can be suppressed, which simplifies the NMR identification and yield quantification of reaction products. Furthermore, this solvent can be readily removed in vacuum. This can be used to concentrate nonvolatile reaction products for spectroscopic observations.

We waited one week until all radical cations decayed (as was indicated by EPR and optical spectroscopies). For preconcentration treatment, this solution was evaporated to dryness and hexane was added. The suspension was sonicated for 5 min and stirred for another 30 min, and LiPF_6_ salt was centrifuged out. The supernate was reduced *in vacuo* and the minimal volume of acetonitrile was added. The reaction products were separated using the same HPLC column (LC-PAH) that we used for quantitative analyses, but we used a 20 μL sample loop instead of a 5 μm loop for perparative work; acetonitrile was used as an eluent. The solvent was removed from the collected fractions, and the residues were dissolved in CDCl_3_ for ^1^H NMR and GCMS characterization. Since NMR signals of these products were still rather weak, necessitating 6-12 h averaging, the acquired NMR spectra were matched against the NMR spectrum obtained from the concentrated solution before fractioning. ^1^H-^1^H COSY and ^13^C NMR spectra were also obtained for the preconcentrated sample before fractioning. To further assist product identification, for **7** we synthesized (following the protocols given in Supplementary Section 1) two isotopomers, in which both of the methoxy groups were deuterated (**7a**) and ^13^C labelled (**7b**).

The absence of proton resonances from **7a** and strong (~140 Hz) proton coupling to ^13^C nuclei in **7b** (resulting in splitting of each proton resonance into a doublet) indicated that the proton in **7** was contained in the former methoxy group. The isotope shifts in the mass spectra indicated how many methoxy groups were contained in a given product. Typically, the reaction yield was too low to observe unlabeled products by ^13^C NMR directly, but for **7b** the signal was sufficiently strong to identify all products containing the methoxy groups even at low yield. DEPT135 sequence was used to distinguish between carbon-13 nuclei coupled to methyl and methylene protons, and WALTZ-256 sequence was used to remove parasitic sidebands.

We first consider **7**, as for this compound the characterization of the reaction products was assisted by the isotope substitution described above. Supplementary Fig. 30a shows the chromatogram of the oxidized solution, and Supplementary Fig. 30b shows the absorption spectra for largest peaks. Both NMR and HPLC indicate that that the recovery of **7** was ca. 47% (which was significantly lower than in PC). Product P1 has the optical band similar to the quinone, and product P2 has a complex spectrum with several absorption bands (whose chemical identity will not be discussed, as they do not occur in carbonate solvents). The product labeled “dimer” in the plot has the absorption spectrum that is notably different from the parent compound (in hindsight, this is due to fusion of benzene rings in **13**). The GCMS of the fraction containing this product shows a single peak corresponding to *m/z* 330 (Supplementary Fig. 31). The fragmentation pattern of this dimer (**13**) is the same as for this species in PC. Our analysis of NMR spectra for **13** gave the following resonances: ^1^H NMR (500 MHz, CDCl_3_) δ 6.74 (s, 2H), 3.78 (s, 6H), 3.39 (s, 6H), 2.26 (s, 6H), 2.19 (s, 6H); ^13^C NMR (125 MHz, CDCl_3_) δ 60.00, 55.35, 12.40, 11.68 (aromatic carbons were not identified). It is easy to see that the only structure that accounts for these mass and NMR spectra is axisymmetric ring-to-ring adduct **13** shown in Supplementary Table 1. This structure is suggested, inter alia, by a greater ^13^C shift and a lower ^1^H shift in out-of-plane methoxy group *c* as compared to in-plane methoxy group *b* (see Supplementary Table 1).

The same type of analyses were carried for other reaction products. We then used NMR spectra to quantify their yields. In this way, we established that the dimer was the main reaction product other than **7** (21.6% yield), whereas strongly absorbing P1 and P2 were, actually, minor products (4.3% and 3.0%, respectively). Thus, in acetonitrile, ring-to-ring radical addition

is the prevalent bimolecular reaction, whereas in PC it was radical disproportionation shown in Fig. 3.

Supplementary Fig. 32a shows the chromatogram of oxidized **7** (before preconcentration) and Supplementary Fig. 32b shows the absorption spectra of the major peaks. Peak P1 originates from quinone **12**. The peak labeled “dimer” in the plot (which has the absorption spectrum similar to that of the parent compound) corresponds to the fraction which yields GCMS chromatogram consisting of a single peak corresponding to *m/z* 330 (Supplementary Fig. 31). The mass spectrum of this dimer (**14**) looks different from **13**: there is a prominent fragment peak at *m/z* 165, indicating that the two monomer units are not connected through the ring (as in **13**), so this ion easily fragments upon the excitation. ^1^H NMR of this product corresponded to δ 6.68 (s, 1H), 6.62 (s, 1H), 6.17 (s, 1H), 3.98 (s, 2H), 3.86 (s, 3H), 3.80 (s, 3H), 3.55 (s, 3H), 3.52 (s, 3H), 2.31 (s, 3H), 2.17 (s, 3H), 1.99 (s, 3H). Again, only one structure can account for these proton NMR and mass spectra: it is **14** shown in Supplementary Table 2. From NMR and HPLC analyses, we found that the recovery of **6** was 67% (which is considerably higher than for **7**) and the yields of **12** and **14** were 14% and 1%, respectively. Thus, for **6**, radical disproportionation was the prevalent bimolecular reaction both in PC and acetonitrile, whereas radical addition with the formation of **14** was negligible, apparently due to the improved steric protection in the arene ring.

**Supplementary Table 1 | ^1^H and ^13^C NMR resonances for dimer 13 generated through the radical addition involving oxidized 6**. ^a^

| **#** | **type** | **δ(^1^H)**  **ppm** | **δ(^13^C)**  **ppm** | ***N*** | **^13^CH_3_** ^b^ | **^13^CD_3_** ^c^ | **^1^H-^1^H**  **COSY** ^d^ |
| --- | --- | --- | --- | --- | --- | --- | --- |
| ***a*** | H-Ar | 6.74 | - | 2 | - | - | b,d |
| ***b*** | MeO | 3.78 | 55.35 | 6 | x | x | a |
| ***c*** | MeO | 3.39 | 60.00 | 6 | x | x | - |
| ***d*** | Me | 2.26 | 11.68 | 6 | - | - | a |
| ***e*** | Me | 2.19 | 12.40 | 6 | - | - | - |

a) chemical shifts in CDCl_3_ vs. TMS, 500 MHz;

b) “x” indicates resonance lines split by 140 Hz (*J*[^1^H-^13^C]) in the methoxy groups (for **7b**);

c) “x” indicates missing proton resonances for **7a**;

d) 2D NMR correlation cross peaks.

**Supplementary Table 2 | ^1^H NMR resonance attribution for dimer 14 generated through the radical addition involving oxidized 7**. ^a^

| **#** | **type** | **δ(^1^H)**  **ppm** | ***N*** |
| --- | --- | --- | --- |
| ***a*** | H-Ar | 6.68 | 1 |
| ***b*** | H-Ar | 6.62 | 1 |
| ***c*** | H-Ar | 6.17 | 1 |
| ***d*** | Ar-CH_2_-Ar | 3.98 | 2 |
| ***e*** | MeO | 3.86 | 3 |
| ***f*** | MeO | 3.80 | 3 |
| ***g*** | MeO | 3.55 | 3 |
| ***h*** | MeO | 3.52 | 3 |
| ***i*** | Me-Ar | 2.31 | 3 |
| ***j*** | Me-Ar | 2.17 | 3 |
| ***k*** | Me-Ar | 1.99 | 3 |

a) chemical shifts in CDCl_3_ vs. TMS, 500 MHz. The C-Ar bond cleavage in the methylene bridge accounts for 165 a.m.u. fragment observed in the mass spectra shown in Supplementary Fig. 31.

**Supplementary Table 3 | Summary of *g*-factors and absolute isotropic hyperfine coupling constants (in Gauss) for protons in radical cations of 6 and 7**.^a^

| Radical cation | (g-2)  x10^4^ | Estimate^a^ | Chemical oxidation^b^ | Electrochemical  oxidation^c^ |
| --- | --- | --- | --- | --- |
| 6^+●^ | 45.1^b^  48.1^c^ | 6H(MeO) 3.79 6H(Me) 4.85 2H 0.12 | 3.16  4.16  1.52 | 3.16  4.17  1.54 |
| 7^+●^ | 44.9^b^  48.1^c^ | 6H(MeO) 3.71 6H(Me) 1.25 2H 2.79 | 2.92  1.57  1.39 | 2.97  1.53  1.32 |

a) The calculations of the hyperfine coupling constants and optimization of radical (ion) structures were carried out using a density functional theory method with the B3LYP functional and 6-31+G(d,p) basis set from Gaussian 09.

b) Chemical oxidation by five equivalents of [bis(trifluoroacetoxy)iodo]benzene in butyronitrile containing five equivalents of trifluoroacetic acid;

c) Electrochemical oxidation of 1 mM solution in PC containing 0.5 M LiTFSI.

**Supplementary Table 4 | Ionic conductivity (at 25 ^o^C) and dynamic viscosity of the electrolyte (at 30 ^o^C) containing compound 6 as a function of the solute concentration.**

| [6], M ^a^ | 0 | 0.05 | 0.1 | 0.15 | 0.2 | 0.25 | 0.3 |
| --- | --- | --- | --- | --- | --- | --- | --- |
| Conductivity  (mS/cm) | 7.68 | 7.31 | 7.57 | 7.62 | 7.63 | 7.52 | 7.54 |
| Viscosity  (cP) | 3.45 | 3.46 | 3.46 | 3.47 | 3.47 | 3.47 | 3.49 |

a) 1.0 M LiTFSI in EC/PC/EMC (4:1:5 by weight).

**Supplementary Figure 1** | **The ^1^H NMR spectrum of 3.**

**Supplementary Figure 2** | **The ^13^C NMR spectrum of 3.**

**Supplementary Figure 3 | The ^1^H NMR spectrum of 4.**

**Supplementary Figure 4** | **The ^13^C NMR spectrum of 4.**

**Supplementary Figure 5** | **The ^1^H NMR spectrum of 5.**

**Supplementary Figure 6 | The ^13^C NMR spectrum of 5.**


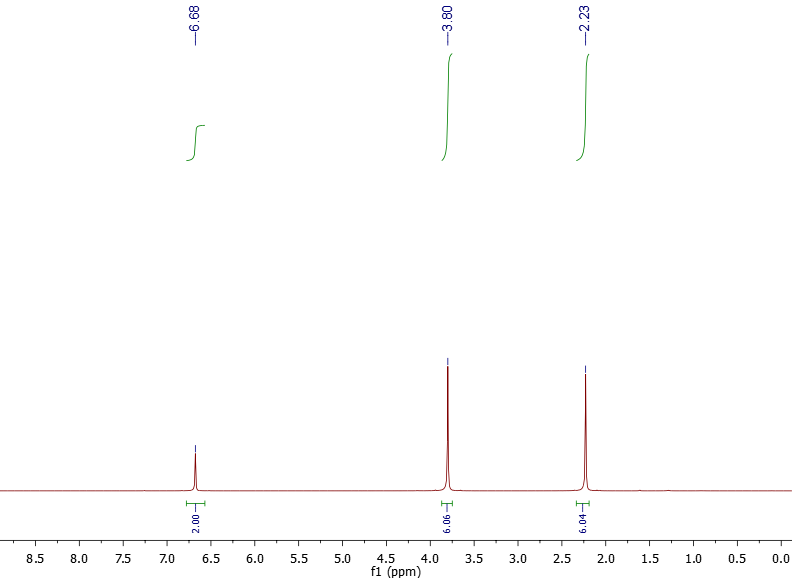


**Supplementary Figure 7 | The ^1^H NMR spectrum of 6.**


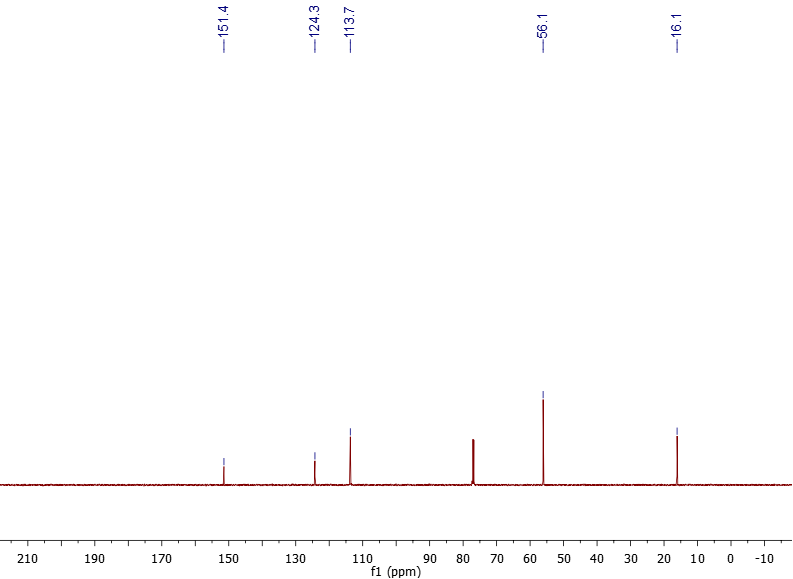


**Supplementary Figure 8 | The ^13^C NMR spectrum of 6.**

**Supplementary Figure 9 |The ^1^H NMR spectrum of 7.**

**Supplementary Figure 10 | The ^13^C NMR spectrum of 7.**

**Supplementary Figure 11 |The ^1^H NMR spectrum of 9.**

**Supplementary Figure 12 | The ^13^C NMR spectrum of 9.**

**Supplementary Figure 13 | The ^1^H NMR spectrum of 10.**

**Supplementary Figure 14 | The ^13^C NMR spectrum of 10.**

**Supplementary Figure 15** | **The ^1^H NMR spectrum of 11.**

**Supplementary Figure 16 | The ^13^C NMR spectrum of 11.**


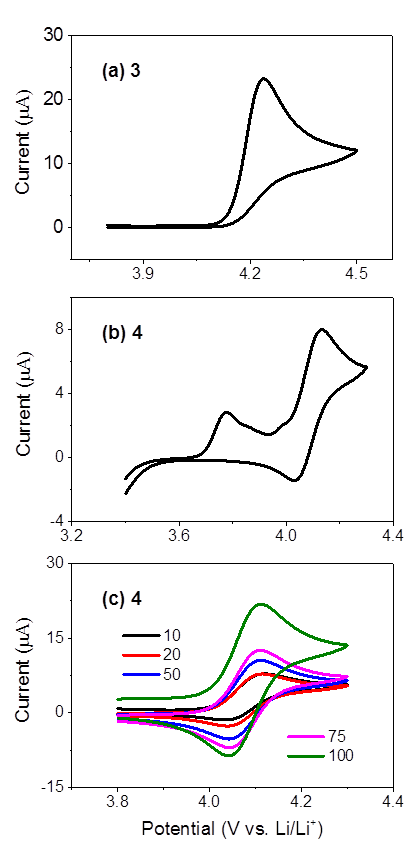


**Supplementary Figure 17** | **Cycling voltammograms for 10 mM solutions of ROMs.** (**a**) **3** at 10 mV/s; (**b**) **4** at 10 mV/s with potential cutoffs at 3.4 V and 4.3 V; (**c**) **4** at various scan rates given in mV/s with potential cutoffs of 3.8 V and 4.3 V.


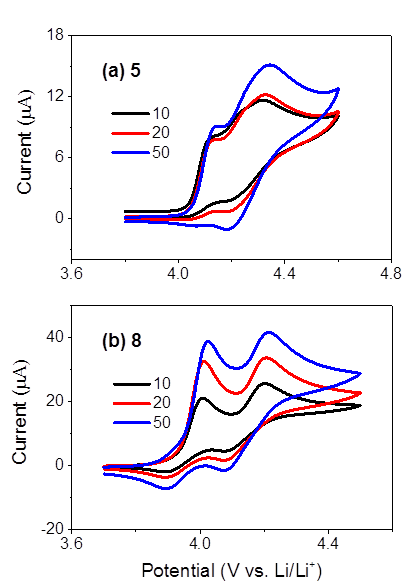


**Supplementary Figure 18 |** **Cycling voltammograms for 10 mM solutions of ROMs.** (**a**) **5** and (**b**) **8** at various scan rates that are given in mV/s in the insets.


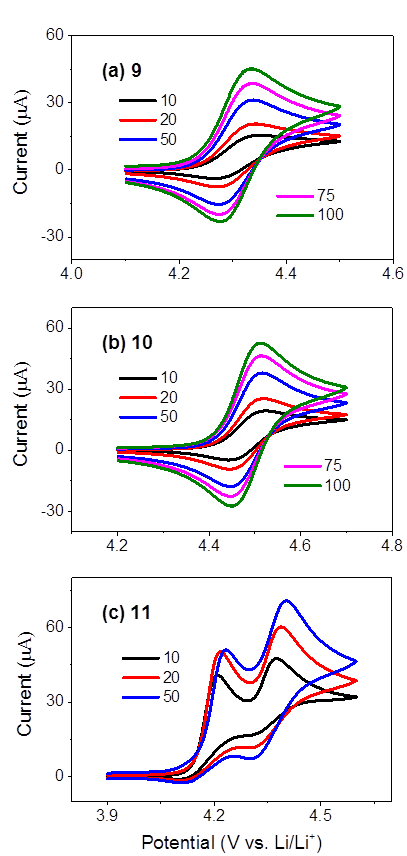


**Supplementary Figure 19** | **Cyclic voltammograms for 10 mM solutions of ROMs.** (**a**) **9**; (**b**) **10**; and (**c**) **11** at various scan rates (in mV/s) that are given in the insets.

**Supplementary Figure 20 | The deprotonation sequence (exemplified for radical cation of 6 in PC).** The reaction is initiated by the deprotonation from the methoxy group with the solvent serving as a proton acceptor. The resulting H loss radical **6**(-H)**^●^** (possibly, in a concerted reaction) abstracts H from the solvent molecule and converts back to **6**. The solvent radicals can release CO­_2_, disproportionate, recombine, or initiate radical polymerization. Since the secondary radicals generated through CO_2_ loss can also abstract H from the solvent, initiating a radical chain reaction, each oxidation event in this diagram can cause significant solvent loss and the accumulation of reaction products that can serve as more efficient proton acceptors compared to the solvent and the solute, accelerating the decay of **6**^+^**^●^**.

**Supplementary Figure 21 | The “color fade” test illustrated for compound 6.** *(To the top)* Absorption spectra obtained by (i) electrochemical oxidation of **6** (from Fig. 3) and (ii) chemical oxidation of **6** in 70 wt% aqueous sulfuric acid. In both cases the absorption is predominantly due to the radical cation **6**^+●^. The corresponding solutions are intensely green. *(To the bottom)* The color fade test, in which 10 vol% of 10 mM **6**^+●^ in the sulfuric acid (see above) was added to (*from left to right*) concentrated sulfuric acid, 2,2,2-trifluoroethanol, acetonitrile, PC, and methanol. The photograph is taken 30 seconds after the addition. The radical cation immediately fades in methanol, but persists in other solvents.


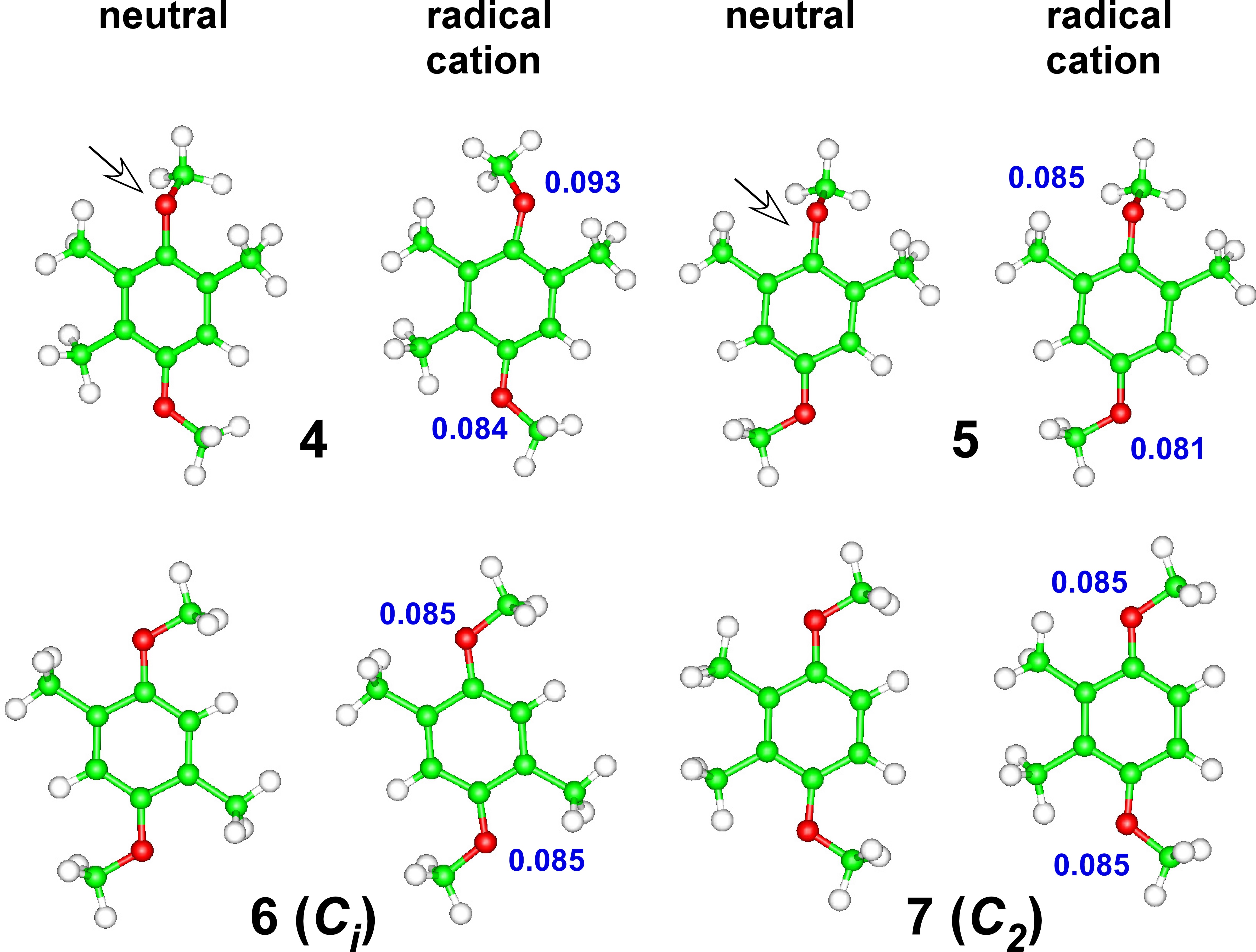


**Supplementary Figure 22 |** **Structural renderings of neutral and radical cation states of ROMs according to density functional theory (DFT) calculations.** The arrows indicate out-of-plane methoxy groups that are forced into this position due to steric hindrance exerted by the two adjacent methyl groups (as there is a high energy barrier for out-of-plane wagging motion of these groups when there is no substitution in the adjacent carbons). In **6^+●^** and **7^+●^**, both of the MeO groups are in the plane of the aromatic ring and are equivalent by axial (or inverse) symmetry, respectively. In **4^+●^** and **5^+●^**, one of MeO groups is out of this plane, and the positive charge in this MeO group is higher than in MeO group that is in-plane, reducing the barrier for deprotonation from the out-of-pane MeO group. The values indicated in the plot give the calculated differences in the Mulliken atomic charges in the oxygen atoms in the charged and neutral state. We caution that DFT generally overestimates the degree of charge localization in asymmetric radical cations as compared to *ab initio* methods (see Supplementary Figure 23).


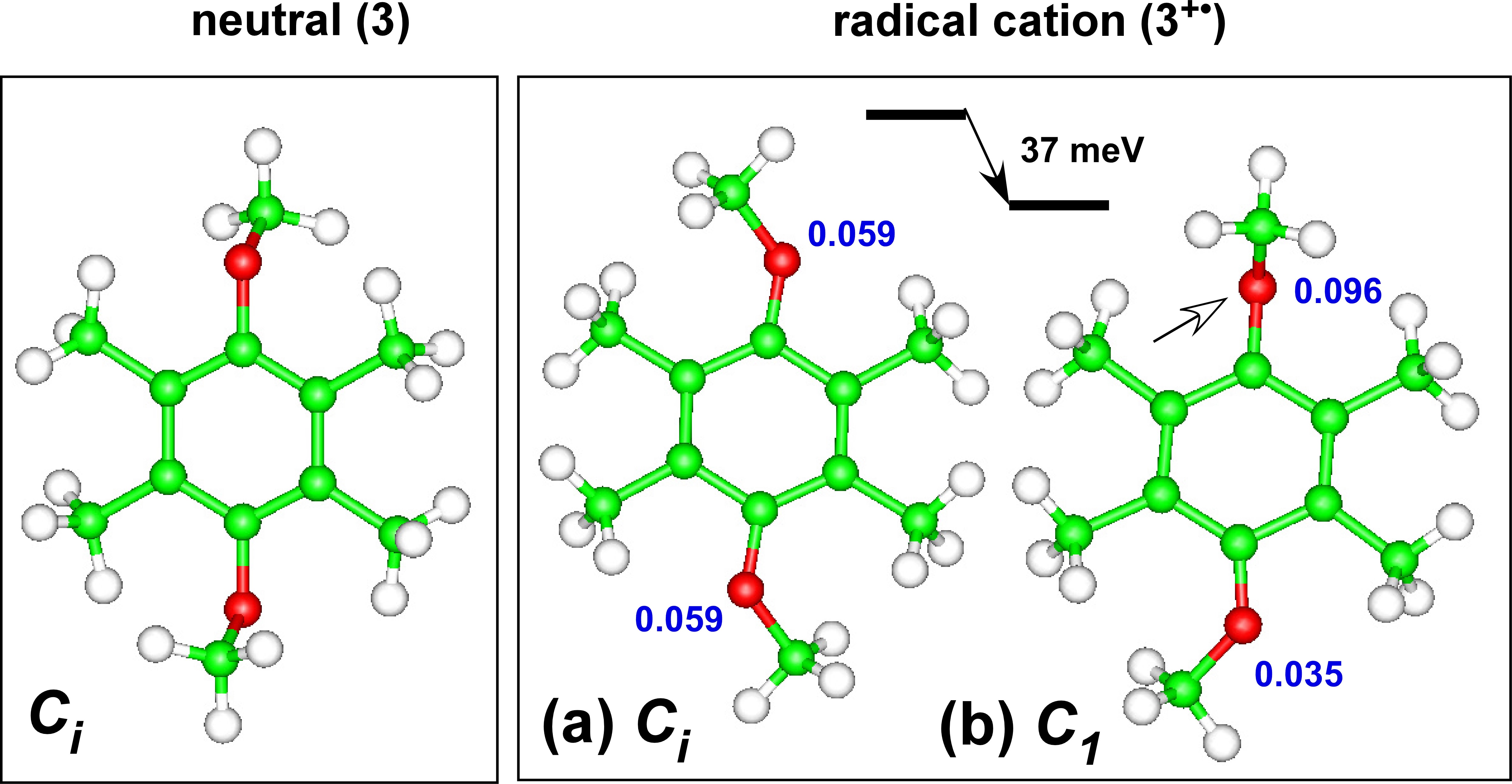


**Supplementary Figure 23 | Structures for neutral and charged states of 3 calculated using unrestricted Hartree-Fock *ab initio* theory.** The neutral form of this molecule is centrosymmetric, but in the radical cation the centrosymmetric conformer *a* is 37 meV higher in energy than broken-symmetry conformer *b*, in which one MeO groups is perpendicular to the aromatic ring (indicated with the arrow), while the second MeO group resides near the plane. The values indicated in the plot give the calculated differences in the Mulliken atomic charges in the oxygen atoms in the charged and neutral states. In the centrosymmetric radical cation *a*, the extra positive charge is partitioned equally between these MeO groups, whereas in the broken symmetry radical cation *b* most of the charge resides in the out-of-plane methoxy group. Unlike the *ab initio* method, the density functional theory predicts conformer *a* as the lowest energy state, overestimating the degree of charge delocalization.

**Supplementary Figure 24 | Like Figure 3 in the text, for electrochemically oxidized 7.**

**Supplementary Figure 25 |** **Quantification of ROM recovery.** The ^1^H NMR spectra from aromatic protons in (**a**) **6** and (**b**) **7** before (trace i) and several days after (traces ii to v) electrolysis of 1 mM solutions in PC containing 0.5 M LiTFSI. The delay times are indicated in the upper panel**.** The chemical shifts are given in ppm *vs*. tetramethylsilane (TMS), and the signals are normalized by the methyl protons in PC. Even two days after the electrolysis the concentration of the radical cations **M^+●^** remained sufficiently high for the degenerate electron exchange, **M** + **M^+●^** ↔ **M^+●^** + **M**, to broaden the resonance line of the parent compounds **M** to zero line (panel b, trace ii). Over a long period of time, [**M^+●^**] gradually decreases so that the resonance lines of **M** become progressively narrower. The NMR spectrum observed 23 days after electrolysis was used to estimate the recovery of the parent compound **M**. To obtain these NMR spectra, an aliquot was diluted 1:4 v/v with CD_3_CN, and benzene was used as the second analytical standard.

**Supplementary Figure 26** | **Absorption spectra of oxidation products.** The species corresponding to 11.7 and 12.8 min peaks in the chromatogram for electrochemically oxidized **6** shown in Supplementary Figure 25a. The 11.7 min species has the absorption spectrum closely resembling of 2,5-dimethoxybenzoquinone (shown in the same plot, compound **12** in Fig. 3), whereas the 12.8 min species has the absorption spectrum identical to **6**. Similar spectra were observed for **7**.


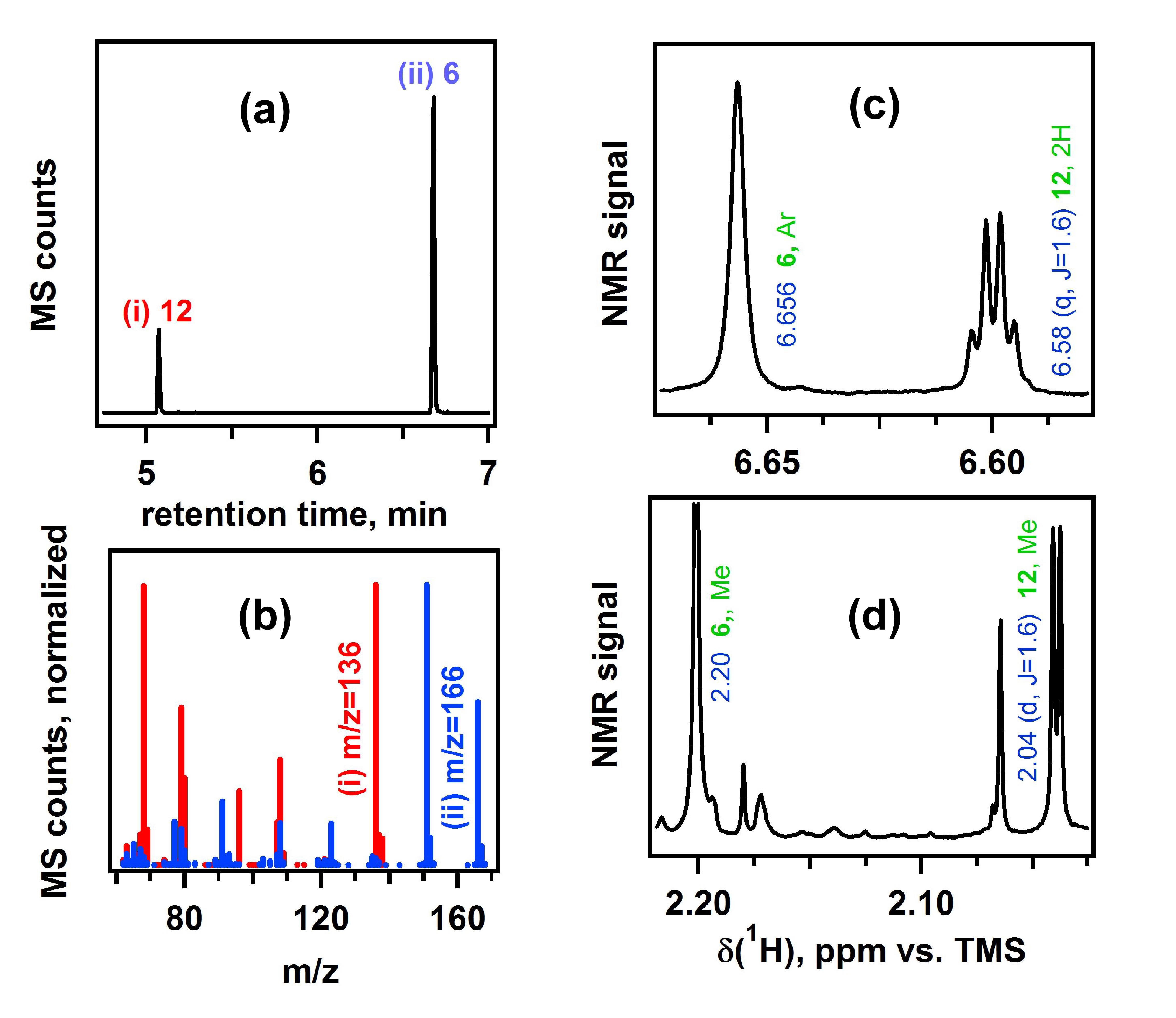


**Supplementary Figure 27** | **Experimental evidence for radical disproportionation.** (**a**) An excerpt of the gas chromatogram, (**b**) mass spectra for the largest two peaks in this chromatogram and (**c,d**) the excerpts of ^1^H NMR spectrum obtained for the concentrated reaction products generated in electrolytic oxidation of **6** (at the completion of the radical cation decay). In the chromatogram, the two main peaks are from (i) 2,5-dimethoxybenzoquinone (product **12**) and (ii) the parent compound (**6**). The corresponding mass spectra (MS) in panel b correspond to *m/z*=136 (**12**) and *m/z*=166 (**6**). In panels (c,d), the proton chemical shifts in CDCl_3_ are given vs. tetramethylsilane (TMS). These panels indicate the resonance lines of **6** and **12** juxtaposed with the lines of the reaction products. The AB system of the aromatic (a quartet) and methyl (a doublet) protons in **12** with 1.6 Hz patterns is observed. Mind that the packed column concentration treatment that we used increases the relative concentration of **12** vs. **6**.

**Supplementary Figure 28 | Chromatograms of oxidized ROMs**. (**a**) **6** and (**b**) **7** obtained shortly after bulk electrolysis. The absorption bands are indicated in the lower panel. The dashed and solid lines correspond to the chromatograms of the solutions obtained before and after the electrolysis, respectively. The two prevalent peaks are from the parent compounds and the related quinones.

**Supplementary Figure 29 | Chromatographic quantification of the quinone yield.** High performance liquid chromatograms of solutions containing (**a**) **6** and (**b**) **7** obtained shortly after electrolysis (black traces) and a week later (red traces) when the radical cations fully decayed. The band pass was 250-255 nm. The concentration of the quinone increased as the reaction progressed. Using the calibrated mixtures, we determined the ratios of the corresponding band-averaged extinction coefficients to be 29.4 for **6** and 34.9 for **7**, respectively. From that, the initial yields of the quinones were estimated to be 4.7% and 5.4% for compounds **6** and **7**, respectively, and the terminal quinone yields were 17.1% and 13.3%, respectively.

**Supplementary Figure 30 | HPLC chromatograms of oxidized 5 mM 6 in acetonitrile containing 0.5 M LiPF_6_.** The solution was oxidized to 100% state-of-charge and analyzed 7 days after the oxidation. (a) Chromatograms for different detection wavelengths indicated in the plot. The dashed trace indicated the chromatogram (288 nm) from the solution before it was oxidized. (b) Absorption spectra for the peaks shown in panel a.

**Supplementary Figure 31 | Mass spectra for *m/z* 330 reaction products 13 and 14 for compounds 6 and 7, respectively.** The arrow indicates the loss of 165 a.m.u. fragment observed in **14** (for **6**) but not in **13** (for **7**).

.

**Supplementary Figure 32 | Like Supplementary Fig. 30, for compound 6.**

**Supplementary Figure 33 | The first-derivative EPR spectra of oxidized ROMs.** Chemically (red traces) and electrochemically (blue traces) oxidized (a) 6 and (b) 7. The spectra at 9.35 GHz were obtained using the modulation frequency of 100 kHz, the microwave field of 2 mW, and the modulation amplitude of 0.2 G. The magnetic field is given in the units of Gauss (1 G = 10-4 T). See Supplementary Table 3 for magnetic parameters.

**Supplementary Figure 34** | **Decay kinetics for radical cation of 6 on the logarithmic vertical scale.** Replotted kinetics given in Figure 4b. The sample temperatures are given in the inset. The initial decay of the radical cation is (pseudo)first order, until ca. 30% of the initial radical cation remains in the solution. As the (secondary) reaction products gradually accumulate they begin to react with the radical cation, and the overall decay accelerates, causing deviations from the straight lines (dashed traces). The initial rate constants given by these lines were used to estimate the activation energy for this reaction given in Supplementary Figure 35.

.

**Supplementary Figure 35 |** **The Arrhenius plot for the initial decay kinetics of 6**^+●^. This plot is for kinetic traces shown in Supplementary Figure 34. Here *k* is the (pseudo)first order rate constant for this exponential decay. The slope of the straight line gives the activation energy for this reaction (in millielectronvolt), which is indicated in the plot. R is the gas constant.

**Supplementary Figure 36** | **The Arrhenius plot for decay kinetics of 7^+●^**. This plot is for kinetic traces shown in Figure 4b. The activation energy for the fast and the slow components of these biexponential kinetics (in millielectronvolt) are indicated in the plot. R is the gas constant. Here *k* stands for the rate constants corresponding to the fast (filled squares) and slow (filled circles) components of the biexponential kinetics. The faster component exhibits a higher activation energy (as seen from the slope of the line) than the slow component.

**Supplementary Figure 37 | The cycling performance of ROMs.** (**a**) Li/**6** and (**b**) Li/**7** flow cells (0.1 M solutions). These plots are identical to Figs. 5c and 5d in the text, respectively, except for using *volumetric energy density* for the left vertical axis. CE, VE, and EE are defined in the text.

**Supplementary Figure 38** | **Rate capability of Li/6 flow cell at 0.1 M at two different current densities.** (**a**) Voltage curves and (**b**) flow cell efficiencies. At higher current, the CE is higher due to shorter cycle time and less facile self-discharge reactions, while the VE is lower due to cell over potential. CE, VE, and EE are defined in the text. The current densities are 5.0 and 7.5 mA/cm^2^. The lower VE at a higher current density is due to the higher cell polarization, which is caused by the increased ohmic and charge transfer resistances. With the rapid electrolyte flow, mass transfer resistance did not have significant impact on this decrease.

**Supplementary Figure 39** | **The efficiencies for Li/6 flow cells.** These cells contained 0.1 M and 0.2 M of **6** in the electrolyte and operated at the current density of 7.5 mA/cm^2^. The flow cell containing more concentrated solution of **6** produced somewhat lower efficiencies. The slightly lower VE is due to the thicker solid electrolyte interphase layer formed on the anode, yielding higher cell resistance, despite the almost identical ionic conductivity and viscosity of the electrolyte at different concentrations of **6** (see Supplementary Table 4). CE, VE, and EE are defined in the text.
